# Arts and creativity and its impact on older people’s health and wellbeing: commentary on a systematic review

**DOI:** 10.12968/pnur.2024.0032

**Published:** 2025-03-02

**Authors:** EJ Sowerby, A Doherty, J Harrison

**Affiliations:** Queen’s Square Medical Practice, Lancaster, UK; University of Lancashire

## Introduction

Globally, many industrialised nations have an ageing population. By the middle of the twenty-first century, those aged 65 years and over are predicted to comprise 16% of the global population (World Health Organisation, 2024). As we age, we may experience a range of risk factors which might adversely impact on our health, wellbeing, and quality of life (Davies et al., 2023; World Health Organisation, 2024). For example, loneliness in older age can be associated with mental ill-health, high blood pressure and heart disease (Guthmuller, 2023). Chronic diseases are also more prevalent in an ageing population (Prince et al., 2015). Alzheimer’s Disease is one of the most common degenerative diseases associated with ageing, affecting over 50 million people worldwide (Alzheimer’s Statistics, 2024). Alzheimer’s Disease is the main cause of dementia, accounting for 60% to 80% of cases (Alzheimer’s Association, 2024). Dementia involves the gradual accumulation of cognitive deficits leading to Mild Cognitive Impairment (MCI), which affects 15.5% of people above the age of 50 worldwide (Bai et al., 2022). Cognitive decline can affect the overall health and well-being of older adults (Salthouse, 2019). The higher disease burden and related costs associated with an increasingly ageing population place pressures on the healthcare systems worldwide (Kehler, 2019). Globally, effective interventions to improve and sustain the health and well-being of ageing populations are urgently needed (Keisari et al., 2024). Thus, there is a call for global action on healthy ageing, especially for sustainable person-centred interventions that are focused on prevention (rather than illness) (World Health Organisation, 2024; Davies et al. 2023).

Engagement in arts and creativity related interventions is receiving attention from governments, industry, philanthropists, policy makers, health professionals, artists, and the general community as part of a healthy ageing strategy (Davies et al. 2023). The World Health Organisation has signified the importance that arts, music and culture can have in promoting health and wellbeing (World Health Organisation, 2011; Fancourt & Finn, 2019). An all-parliamentary UK enquiry suggested that arts and creative interventions may keep people well and support `longer lives that are better lived’ (All-Party Parliamentary Group, 2017; Gordon-Nesbitt & Howarth, 2020). Such interventions are thought to delay cognitive decline and improve physical and mental health and wellbeing outcomes for older people – including those with mild cognitive impairment (Fong et al., 2021; Lin et al., 2022) and depression (Zhao et al., 2016).

### Aim of commentary

The review conducted by McQuade and O’Sullivan (2023) aimed to examine the impact of participation in group-based arts and creativity interventions on physical and psychosocial health and wellbeing outcomes in older adults (aged 50 years and above). This commentary critiques and considers the review’s results alongside related research. The commentary may provide insights for the future development of arts and creativity preventative policy and practice, with the latent potential to improve a range of physical and mental healthcare outcomes for ageing populations.

### Methods used by McQuade and O’Sullivan (2023)

The review authors used the Preferred Reporting Items for Systematic Reviews and Meta-Analysis (PRISMA) guidelines to help synthesise the available evidence (Moher et al., 2009). The review protocol was registered with PROSPERO, the International Prospective Register of Systematic Reviews (CRD42021234832). An extensive range of databases were checked from a variety of disciplines, using a predefined search strategy. The search was limited to a seven-year period, January 2013 to December 2020. The time limitation reason given by the authors was that creative arts is an emerging discipline. Full text, empirical studies published in the English Language were included, with inclusion of studies based on older adults defined as 50 years or older. The age-range was chosen by the authors to reflect the worldwide trajectory of health and ageing across the life course. Two reviewers screened the titles and abstracts of studies from the varied databases for eligibility as well as full text articles. Data extraction was carried out by one reviewer using a pre-piloted data extraction form and validated by a second reviewer. For studies meeting the eligibility criteria, two reviewers used a Mixed Methods Appraisal Tool (MMAT) (Hong et al., 2018) to critically appraise the literature.

### Results of McQuade and O’Sullivan (2023)

The review by McQuade and O’Sullivan (2023) analysed 93 included studies from 21 different countries. Results by McQuade and O’Sullivan (2023) are summarised in Table 1.

### Commentary

A critical appraisal of the 2023 review by McQuade and O’Sullivan was undertaken by two independent reviewers using the JBI critical appraisal tool for assessing systematic reviews (Aromataris et al., 2015). The review met eight out of eleven items in the tool’s criteria for assessment (see Table 2). The quality assessment highlighted that although a comprehensive and systematic literature search was undertaken - including a search of grey literature - there was no justification provided as to why only studies published in English were included. Whilst the review states that two reviewers assessed studies’ risk of bias it is unclear whether they did this independently of each other. Furthermore, only one reviewer performed data extraction which was then validated by a second reviewer. Recognised systematic review (PRISMA) guidelines recommend that at least two independent reviewers should undertake the screening, quality assessment and data extraction stages of a review (Moher et al., 2009). The review’s authors did provide specific directives for new research based on their findings. However, no clear recommendations for policy and practice supported by their findings were made explicit.

Overall, McQuade and O’Sullivan’s findings suggest that participating in group-based arts and creativity interventions may have positive physical and mental health benefits for older adults. However, some caution should be applied when interpreting the review’s results due to limitations. For example, the types of studies, interventions, their duration, outcomes assessed, and results varied. Furthermore, the review’s search was limited to studies published in the English Language (only) within a 7-year period; and most of the studies included small sample sizes of healthy, mostly female, older adults (defined as being aged 50 years and above). Despite this, findings from other reviews and sources of evidence appear to be consistent with the findings of McQuade and O’Sullivan (2023). For example, a review conducted for the UN/WHO’s `*Decade of Action for Healthy Ageing for All*’ suggested that music and dance may make a positive contribution to healthy ageing; and are likely to have high initial and ongoing use because individuals are more likely to do activities which are fun, easy, low cost or free, portable, and culturally acceptable (Shaffer, 2022). Similarly, a qualitative systematic review found that music & dance has the potential to be an effective and sustainable community-based activity that contributes significantly to healthy active ageing, but that there are several gaps in the literature including the under-representation of male participants, indigenous participants, LGBTQIAP+ participants, and participants from migrant populations (Sheppard & Broughton, 2020). In terms of active musical participation, findings from two recent systematic reviews suggest beneficial effects on cognitive and psychosocial functioning (Vetere et al., 2024; Viola et al., 2023). Likewise, a recent review of group-based music-based interventions suggests that they may have positive effects on health outcomes, but that some studies yielded inconclusive or no effects, and most were less than six months in duration with the longer-term effects not being fully determined (Ma and Ma, 2023). Comparably, dancing as an intervention may provide effective and enjoyable `exercise in disguise’ for older people with diverse mobility profiles, and contributes to healthy, happy ageing according to a recent realist review (Haynes et al., 2023). Some emerging primary studies (including randomised controlled trials [RCTs]) also suggest that group-based singing interventions are associated with positive effects on individuals’ general and perceived health (Kaasgaard et al., 2022; Galinha et al., 2021a; Galinha et al., 2021b). Furthermore, a critical realist review found that place-based creative programmes such as music making, singing, visual arts making, and painting which are usually run in community spaces, impact positively on older people’s mental, social, and physical health (Bellazzecca et al., 2022).

### Further research recommendations

Further robust research is required to fully understand the benefits of different types of group-based arts and creativity interventions for the promotion of health and wellbeing and the prevention of ill-health in later life for diverse population groups. Researchers should apply a health equity lens to identify and explore intersectionality issues for diverse populations in future intervention studies. Resources are available to support researchers and research advisers to better understand how to embed equality, diversity, and inclusion (EDI) into research design and implementation (NIHR EDI Toolkit; FOR-EQUITY – tools and resources to help reduce social and health inequalities).

Future studies may usefully consider the optimal duration, type, delivery methods, frequency, intensity and costs of their interventions for diverse population groups and assessment of longer-term effects.

### Implications for policy and practice

There is scope for inter-disciplinary collaborations, working with diverse communities, to co-develop safe, effective, and tailored arts and creativity-related interventions to enhance the health and wellbeing of diverse population groups. Arts and creativity-related interventions may be considered as (preventative-focused) health promotion or social prescribing resources (NHS 2021; Local Government Association, 2016; Public Health England 2016).

Social prescribing is a process whereby healthcare professionals such as practice nurses, doctors, or social workers, refer people to non-medical services to improve their health and wellbeing (Office for Health Improvement & Disparities, 2022). Social prescribing connects individuals with relevant community-based resources, networks, and non-pharmacological activities – such as arts and creativity-related interventions (NHS England 2024). The social prescribing approach recognises that health and wellbeing are influenced by a wide range of social, economic, cultural, and environmental factors, not just physical or medical conditions (Husk et al., 2020). For example, dance in the Western world is often stereotypically associated with women which can make it challenging for men to enter the world of dance even for health and wellbeing purposes (Mennesson, 2009). Familiarity with the local community, and its transport links, social networks and peer support are all important considerations: e.g., a man’s willingness to attend a dance class in a local community centre along with his partner may increase if he learns that some of his male friends already attend with their partners (McKinnon, 2006).

The review by McQuade & O’Sullivan (2023) found a lack of longer-term funding for arts and creativity-related interventions and a need for a more integrated public health and arts policy approach. Economic evaluations of arts and creativity-related interventions are needed to support policy and decision-making investments for future funding and commissioning purposes.

#### Practice Nurses

Practice nurses in primary care support people’s health and wellbeing through person-centred care approaches which consider the ‘person’ as opposed to the ‘patient’; and its planning offers people active involvement in decisions about how their health will be managed (NHS England, 2018). Practice nurses are similarly familiar with the concept of community referrals and with the opportunities that social prescribing can provide for personalised care through salutogenic principles^*^ as promoted by the NHS Long-Term Plan’s comprehensive personalised care model (NHS, 2019). Practice nurses are well-placed as part of the practice team to be able to encourage patients to access a range of social prescribing activities including arts and creativity-related interventions. Interdisciplinary partnership teams could, potentially, help co-design arts and creativity-related interventions to meet the preventative health needs of their diverse communities. However, with increasing workloads and competing priorities in health and social care practice - and in other public sector partner organisations such as local authorities - this may prove challenging.

## Conclusions

Overall, emerging evidence suggests that group-based arts and creativity interventions may have the potential to positively impact on people’s health, wellbeing, and quality of life. However, there is limited robust evidence available including a lack of studies involving diverse population groups; and the costs and longer-term benefits and sustainability of several types of interventions for different population groups needs further exploration.

### CPD reflective questions (insert 1 – 5 questions)

1. What are the main limitations of the review by McQuade and O’Sullivan 2023?
2. What future research is required in this area?
3. What developments are needed to help shape more inclusive arts and creativity-related interventions that meet the health and wellbeing needs of diverse population groups e.g., older men as well as women, people from different ethnic minorities, people with intellectual disabilities, migrant workers, homeless people, persons who identify as LGBTQIAP+, people from different faith groups / religions or with different beliefs, people living in poverty?
4. How can those working in health and social care (and other public sector partners such as local government) go about modifying their working patterns to focus more on preventative health as promoted by the NHS’ Long-term Plan?
5. How do we develop arts and creativity-related partnerships, and embed the arts and creative health agenda outside the four walls of a primary care practice?

## Figures and Tables

**Table 1:** Summary of results by McQuade and O’Sullivan (2023)

Table 1: Characteristics of studies included in the review by McQuade and O’Sullivan (2023)	
**Population**	Cumulative total of 7927 participants aged between 50 - 94 years. Studies’ sample sizes varied (from four participants in a qualitative study to 750 participants in a case-controlled study). Most studies included healthy older adults (n=82), and some included diagnosed health conditions (n=11).
**Study designs**	Quantitative (n=57); qualitative (n=19), mixed methods (n=17).
**Interventions**	Dance was the most common group-based arts and creativity intervention assessed (n=44 studies), followed by music & singing (n=34), visual & creative arts (n=12), and drama & theatre (n=3).
**Outcomes**	The review’s studies examined a range of different health and wellbeing outcomes using different outcome measures.
**Summary of intervention outcomes:**	
**Dance (n=44 studies)**	
McQuade & O’Sullivan (2023) concluded that there was convincing evidence to suggest that dance is associated with a positive impact on older adults’ physical health and wellbeing including: improvements in balance & mobility, and physical fitness outcomes. They also commented that there was promising evidence suggesting benefits associated with dance and improvements in brain function, cognitive function, and body composition. However, evidence was limited or inconsistent for improvements associated with independent living, risk of falling, and psycho-social outcomes.	
**Music & Singing (n=34 studies)**	
McQuade & O’Sullivan (2023) found only limited evidence to suggest any improvements in physical health outcomes associated with music and singing interventions, but some promising evidence to suggest improvements in psychological, social health and wellbeing outcomes.	
**Visual & Creative Arts (n=12 studies)**	
The review authors found limited evidence to suggest any physical or psychological health and wellbeing benefits associated with visual and creative arts interventions. However, they suggested that there was promising evidence to suggest that these interventions improved participants’ sense of general wellbeing.	
**Drama & Theatre (n=3 studies)**	
The review authors found no evidence to suggest that drama & theatre interventions were associated with improvements in physical health and wellbeing outcomes, and only limited evidence of some form of impact on psycho-social improvement outcomes.	

**Table 2:** Quality Assessment (using JBI critical appraisal checklist for systematic reviews*)

1. Is the review question clearly and explicitly stated?	Yes. The review comprised four research questions, formulated around their PICO (Population, Intervention, Comparator, Outcome). These questions were set out in the review’s protocol.
2. Were the inclusion criteria appropriate for the review question?	Yes. The inclusion criteria matched the review’s questions.
3. Was the search strategy appropriate?	Yes. Fourteen relevant electronic databases were searched using predefined search criteria developed by the researchers in consultation with a Subject Librarian. A combination of Medical Subject Headings (MeSH), keywords and free-text search terms were employed and modified for each electronic database. The search strategy was included in the supplementary data.
4. Were the sources and resources used to search for studies adequate?	Yes. Multiple electronic databases were searched. Grey literature searches were also included.
5. Were the criteria for appraising studies appropriate?	Yes. Studies were critically appraised using the Mixed Methods Appraisal Tool (MMAT) (Hong et al., 2018).
6. Was critical appraisal conducted by two or more reviewers independently?	Unclear. The review states that the studies were critically appraised by two reviewers. However, it is unclear from the publication or from the review’s protocol whether the two reviewers did this independently from each other.
7. Were there methods to minimize errors in data extraction?	Unclear. Data extraction was undertaken by one reviewer using a pre-piloted data extraction form and validated by a second reviewer. However, PRISMA guidelines recommend that this process is conducted by two independent reviewers to minimize bias.
8. Were the methods used to combine studies appropriate?	Yes. A narrative synthesis was conducted due to the heterogeneity of included studies.
9. Was the likelihood of publication bias assessed?	Yes. Comprehensive search strategy conducted to alleviate the impact of publication bias.
10. Were recommendations for policy and/or practice supported by the reported data?	Unclear.
11. Were the specific directives for new research appropriate?	Yes. The authors recommend further research across a range of various types of arts and creativity interventions is needed to develop a greater understanding of the evidence on health benefits for older adults.

* *Ref:* Aromataris et al., 2015

## Key points

- Arts and creativity-related interventions are receiving global attention from governments, policymakers, and health professionals as part of their healthy ageing strategies.
- Emerging evidence suggests that participating in dance may have positive physical and mental health outcomes for mainly healthy, female older adults aged 50 years and above.
- There is scope for inter-disciplinary collaborations, working with diverse communities, to co-develop safe, effective, and tailored arts and creativity-related interventions to enhance the health and wellbeing of diverse population groups.
- Further research is needed to ascertain the clinical and cost effectiveness of different types of arts and creativity-related interventions for our diverse communities, including the longer-term benefits and sustainability of the interventions.

## Footnotes

* Salutogenesis is an asset-based approach which focuses on factors that support an individual’s health and wellbeing, rather than on factors that cause disease (pathogenesis).
